# Experimental data on surface roughness and force feedback analysis in friction stir processed AA7075 – T651 aluminium metal composites

**DOI:** 10.1016/j.dib.2019.103710

**Published:** 2019-03-08

**Authors:** Omolayo M. Ikumapayi, Esther T. Akinlabi

**Affiliations:** Department of Mechanical Engineering Science, University of Johannesburg, Auckland Park Kingsway Campus, Johannesburg, 2006, South Africa

**Keywords:** Friction stir processing, Surface roughness analysis, Reinforcements, Force feedback

## Abstract

Friction Stir Processing (FSP) is a surface modification technique used to enhance the mechanical properties and improve the surface integrity of the processed material. In the present data collection, aluminium alloy 7075-T651 was studied under different reinforcement conditions. Microchannel of dimension 3.5 mm depth and 2.0 mm width were machined on the aluminium plates to accommodate the particles. The process was conducted at different rotational speed of 1200 rpm, 1500 rpm and 1800 rpm with constant processing speed of 20 mm/min, plunge depth of 0.3 mm and tilt angles of 3°. Double passes were achieved for each parameter with 100% inter-pass overlap. A cylindrical tapped, AISI H13 steel tool with shoulder diameter 18 mm, pin length of 5.0 mm, pin diameter 5 mm at the top and 6 mm at the end with 10° taper was used during friction stir process. Surface integrity analysis was carried out with the aid of mitutoyo surftest SJ-210 surface roughness tester (SRT). The analysis was carried out at three different points on a parameter for a particular workpiece and the average reading for each parameter is calculated in order to ensure precision of the measurements and the coverage surface area. The following surface roughness parameters were measured and recorded, arithmetical mean roughness value (Ra), maximum height (Ry), mean roughness depth (Rz) and root mean square roughness (Rq). Force feedback from the machine data for selected reinforcement particles with respected to processing times and x-positions are also presented.

**Table:** Specifications table

Subject area	*Mechanical Engineering & Material Science*
More specific subject area	*Friction Stir Processing and Surface Engineering*
Type of data	*Tables and Graphs*
How data was acquired	*Experimental data in the tables were acquired through surface roughness measurement of the processed aluminium metal composites. The graphs were acquired during processing operation from the force feedback on the workpiece being process.*
Data format	*Raw, and Analysed*
Experimental factors	*Microchannel of dimension 3.5* *mm depth and 2.0* *mm width were machined on the 7075-T651 aluminium plates to accommodate the particles. The process was conducted at different rotational speed of 1200 rpm, 1500 rpm and 1800 rpm with constant processing speed of 20* *mm/min, and plunge depth of 0.3* *mm. Double passes were achieved for each parameter with 100% inter-pass overlap. A cylindrical tapped, AISI H13 steel tool was used to carry out the FSP experiments with 18* *mm shoulder diameter, pin length of 5.0* *mm, and pin diameter 5* *mm at the top and 6* *mm at the end. The Surface roughness analysis was carried out at three different points on a processed parameter.*
Experimental features	*FSP was carried out at room temperature along initial rolling direction of the plate. The tool tilt angle was kept constant at 3 ° throughout the operations relative to the normal direction of the plate surface.*
Data source location	*Indian Institute of Technology, Kharagpur, INDIA*
Data accessibility	*The availability of the dataset is within the peripheral of this article*

**Table:** 

**Value of the data** • The FSP parameters presented here is an optimum parameters that give defect free zone and provide processing window for various second phases into AA7075-T651 to make composites • Similar optimum processing parameters can be applied to other metal matrix composites • Reported process parameters will help and guide other researchers to select suitable process parameters for AA7075-T651 aluminium alloy without going through rigorous trials and errors. • The reported force feedback data will help the future researchers to know how the surface finish of their processing plate will look like when applying the parameters as well as the reinforcements. • Chemical compositions and the mechanical properties of AA7075-T651 will be of valuable data for future users.

## 1 Data

The Data presented are from the characterization of friction stir processed of Aluminium Alloy 7075-T651 with different reinforcement agro-waste powders such as coal fly ash (CFA), wood fly ash (WFA) [1], cow bone ash (CBA) [2], coconut shell ash (CSA) [3], palm kernel shell ash (PKSA) [4]; and metallic powders such as stainless steel alloy powder (17-4Ph) and titanium alloy powder (Ti-6Al-2Sn-2Zr-2Mo-2Cr-0.25 Si). The agro-waste powders were firstly milled into nanoparticle individually and then pour inside pure graphite crucible and heat treated in a muffle furnace set at 500 °C for 1 hour and then cooled inside the furnace to room temperature before applying it on the substrate for processing. The chemical composition of the parent metal is presented in Table 1 while the Mechanical properties are presented in Table 2. The process parameters used in the data collection of the frictions stir processed of Aluminium Alloy 7075-T651are in Table 3 the variable parameter is tool rotational speed and reinforcement phases; and the constant parameters are processing speed, tool tilt angle, and plunge depth. Dataset for the tool design used also presented. Data from Surface roughness Tester for the processed samples for the prediction of surface integrity were also presented in Table 4, Table 5, Table 6, Table 7, Table 8, Table 9, Table 10, Table 11, Table 12, Table 13, Table 14. Data from the force feedback on the processed samples are also presented in (Fig. 7, Fig. 8, Fig. 9, Fig. 10, Fig. 11, Fig. 12, Fig. 13, Fig. 14). Various equipment used and their methods for data collections are also presented.

**Table 1:** Chemical composition of the parent metal used (AA7475 – T651).

Elements	Si	Fe	Cu	Mn	Cr	Zn	Ti	Mg	Al
Wt.% composition	0.05	0.15	1.93	0.01	0.193	5.92	0.02	2.8	Bal

**Table 2:** Mechanical Properties of the parent metal used (AA7475 – T651).

Properties	Value
Ultimate tensile strength (UTS)	570 MPa
Yield strength	500 MPa
Shear strength	330 MPa
Shear modulus	26 GPa
Fatigue strength	160 MPa
Elastic modulus (young's, tensile)	70 GPa
Poisson's ratio	0.32
Brinell hardness	150
Elongation at break	8.2%

**Table 3:** Friction stir processing parameters (variable and constant).

S/N	Process parameter	Value
1	Processing speed (mm/min) – pinless (probeless)	100
2	Rotational speed (rpm) – pinless (probeless)	1000
3	Processing speed (mm/min) – tool with pin	20
4	Rotational speed (rpm) – tool with pin	1200, 1500, 1800
5	Tool tilt angle (°)	3
6	Tool penetration (plunge) depth (mm)	0.3
7	Pass (no)	2
8	Plunge rate (mm/min)	30
9	Processing configuration	Position controlled
Tool design		
1	Tool material	H13 Hot-working tool steel
2	Tool shoulder diameter (mm)	18
3	Tool pin diameter (mm)	5
4	Tool pin length (mm)	5
5	Tool pin profile (shape), outer surface	Cylindrical
6	Tool pin profile (shape), end surface	tapered
Groove design		
1	Plate dimensions (mm^3^)	300 × 125 x 6
2	Groove width (mm)	2
3	Groove depth (mm)	3.5
4	Groove length (mm)	280

**Table 4:** Experimental data of surface roughness analysis for friction stir processed base metal (AA7075-T651).

AA7075-T651										
Rotational speed (rpm)	Processing speed (mm/min)	Point of measurement	Ra (μm)	Mean Ra (μm)	Ry (μm)	Mean Ry (μm)	Rz (μm)	Mean Rz (μm)	Rq (μm)	Mean Rq (μm)
1200	20	P1	11.34	11.55	75.26	75.67	58.84	59.24	14.16	14.27
		P2	11.67		76.02		59.68		14.46	
		P3	11.65		75.74		59.20		14.20	
1500	20	P1	12.05	11.61	78.87	76.65	60.92	59.47	15.80	14.62
		P2	11.24		75.11		58.42		14.02	
		P3	11.53		75.98		59.07		14.05	
1800	20	P1	9.11	11.05	63.86	72.44	47.19	57.27	11.49	13.41
		P2	10.20		65.39		53.23		12.40	
		P3	13.84		88.07		71.28		16.33

**Table 5:** Experimental data of surface roughness analysis for friction stir processed AA7075-T651/CFA matrix composites.

AA7075-T651/CFA										
Rotational speed (rpm)	Processing speed (mm/min)	Point of measurement	Ra (μm)	Mean Ra (μm)	Ry (μm)	Mean Ry (μm)	Rz (μm)	Mean Rz (μm)	Rq (μm)	Mean Rq (μm)
1200	20	P1	5.00	5.20	39.82	37.10	27.82	26.48	6.42	6.47
		P2	3.97		23.93		21.04		4.51	
		P3	6.62		47.50		30.58		8.48	
1500	20	P1	6.88	7.50	48.23	52.69	30.31	37.79	8.58	8.86
		P2	8.40		59.71		44.99		9.12	
		P3	7.23		50.12		38.06		8.90	
1800	20	P1	4.99	5.76	43.08	45.20	28.16	35.56	6.68	9.75
		P2	6.75		49.89		48.06		14.45	
		P3	5.53		42.52		30.47		8.13

**Table 6:** Experimental data of surface roughness analysis for friction stir processed AA7075-T651/CBA matrix composites.

AA7075-T651/CBA										
Rotational speed (rpm)	Processing speed (mm/min)	Point of measurement	Ra (μm)	Mean Ra (μm)	Ry (μm)	Mean Ry (μm)	Rz (μm)	Mean Rz (μm)	Rq (μm)	Mean Rq (μm)
1200	20	P1	2.31	2.00	20.44	17.53	11.05	10.78	3.28	2.70
		P2	1.85		19.86		10.52		2.60	
		P3	1.82		12.29		10.78		2.23	
1500	20	P1	2.87	2.81	19.84	20.24	14.31	14.82	3.68	3.56
		P2	2.89		20.56		15.42		3.53	
		P3	2.68		20.31		14.75		3.46	
1800	20	P1	2.79	2.36	19.95	17.24	14.40	13.05	3.64	3.08
		P2	2.24		14.57		12.53		2.78	
		P3	2.06		17.21		12.22		2.82

**Table 7:** Experimental data of surface roughness analysis for friction stir processed AA7075-T651/WFA matrix composites.

AA7075-T651/WFA										
Rotational speed (rpm)	Processing speed (mm/min)	Point of measurement	Ra (μm)	Mean Ra (μm)	Ry (μm)	Mean Ry (μm)	Rz (μm)	Mean Rz (μm)	Rq (μm)	Mean Rq (μm)
1200	20	P1	2.51	2.48	24.95	24.42	16.43	16.00	3.88	3.72
		P2	2.76		25.81		16.79		3.96	
		P3	2.17		22.52		14.79		3.32	
1500	20	P1	2.13	1.60	16.97	12.63	14.55	9.99	2.66	1.98
		P2	1.17		9.89		6.30		1.47	
		P3	1.48		11.02		9.12		1.83	
1800	20	P1	1.78	1.92	14.83	19.71	11.43	11.83	2.26	2.59
		P2	1.49		20.65		9.38		2.13	
		P3	2.48		23.64		14.69		3.38

**Table 8:** Experimental data of surface roughness analysis for friction stir processed AA7075-T651/CSA matrix composites.

AA7075-T651/CSA										
Rotational speed (rpm)	Processing speed (mm/min)	Point of measurement	Ra (μm)	Mean Ra (μm)	Ry (μm)	Mean Ry (μm)	Rz (μm)	Mean Rz (μm)	Rq (μm)	Mean Rq (μm)
1200	20	P1	2.67	2.47	23.60	23.92	14.67	14.63	3.79	3.33
		P2	2.73		28.76		15.98		3.61	
		P3	2.01		19.39		13.23		2.58	
1500	20	P1	3.00	2.98	27.03	26.10	19.73	19.19	3.88	3.88
		P2	3.01		28.61		20.69		3.96	
		P3	2.93		22.65		17.17		3.79	
1800	20	P1	3.38	2.76	24.67	22.35	20.01	17.57	3.76	3.35
		P2	2.09		20.23		14.95		2.95	
		P3	2.80		22.16		17.75		3.33

**Table 9:** Experimental data of surface roughness analysis for friction stir processed AA7075-T651/PKSA matrix composites.

AA7075-T651/PKSA										
Rotational speed (rpm)	Processing speed (mm/min)	Point of measurement	Ra (μm)	Mean Ra (μm)	Ry (μm)	Mean Ry (μm)	Rz (μm)	Mean Rz (μm)	Rq (μm)	Mean Rq (μm)
1200	20	P1	4.89	5.32	50.23	65.91	25.76	27.29	9.42	10.04
		P2	5.80		82.20		28.76		10.93	
		P3	5.27		65.30		27.36		9.76	
1500	20	P1	5.10	4.43	62.87	44.56	29.61	24.82	7.80	6.38
		P2	2.91		25.01		16.07		4.06	
		P3	5.30		45.79		28.78		7.29	
1800	20	P1	5.69	5.20	52.49	50.28	32.17	29.24	9.99	9.49
		P2	5.23		47.64		28.71		9.71	
		P3	4.68		50.70		26.84		8.76

**Table 10:** Experimental data of surface roughness analysis for friction stir processed AA7075-T651/Stainless Steel (17-4Ph) matrix composites.

AA7075-T651/Stainless steel (17-4Ph)										
Rotational speed (rpm)	Processing speed (mm/min)	Point of measurement	Ra (μm)	Mean Ra (μm)	Ry (μm)	Mean Ry (μm)	Rz (μm)	Mean Rz (μm)	Rq (μm)	Mean Rq (μm)
1200	20	P1	14.00	12.81	83.34	76.90	68.18	66.26	15.06	13.53
		P2	12.01		70.45		62.77		12.56	
		P3	12.41		76.91		67.84		12.98	
1500	20	P1	12.19	11.67	71.24	68.81	60.62	57.31	15.39	13.41
		P2	11.61		69.31		56.77		12.55	
		P3	11.20		65.89		54.53		12.30	
1800	20	P1	12.13	12.02	76.20	74.26	61.08	59.56	16.10	15.96
		P2	11.55		67.97		54.96		14.00	
		P3	12.39		78.62		62.63		17.77

**Table 11:** Experimental data of surface roughness analysis for friction stir processed AA7075-T651/Ti-6Al-2Sn-2Zr-2Mo-2Cr-0.25 Si matrix composites.

AA7075-T651/Ti-6Al-2Sn-2Zr-2Mo-2Cr-0.25 Si										
Rotational speed (rpm)	Processing speed (mm/min)	Point of measurement	Ra (μm)	Mean Ra (μm)	Ry (μm)	Mean Ry (μm)	Rz (μm)	Mean Rz (μm)	Rq (μm)	Mean Rq (μm)
1200	20	P1	2.29	2.67	21.49	20.72	15.08	16.82	3.02	3.66
		P2	3.43		24.60		21.81		4.38	
		P3	2.31		16.08		13.59		3.59	
1500	20	P1	2.79	3.05	21.73	22.65	16.38	19.00	3.83	4.08
		P2	3.22		23.88		20.00		4.34	
		P3	3.13		22.35		20.63		4.09	
1800	20	P1	3.04	3.02	23.86	28.14	20.60	21.12	3.90	3.97
		P2	2.61		36.59		19.44		4.03	
		P3	3.42		23.96		23.33		3.99

**Table 12:** Experimental data of surface roughness analysis for friction stir processed AA7075-T651/WFA/Ti-6Al-2Sn-2Zr-2Mo-2Cr-0.25 Si hybrid composites.

AA7075-T651/WFA/Ti-6Al-2Sn-2Zr-2Mo-2Cr-0.25 Si										
Rotational speed (rpm)	Processing speed (mm/min)	Point of measurement	Ra (μm)	Mean Ra (μm)	Ry (μm)	Mean Ry (μm)	Rz (μm)	Mean Rz (μm)	Rq (μm)	Mean Rq (μm)
1200	20	P1	9.55	9.40	95.34	95.41	55.34	54.26	14.64	13.35
		P2	9.74		97.28		56.59		15.20	
		P3	8.91		93.61		50.87		13.22	
1500	20	P1	9.80	9.27	95.76	95.67	55.45	54.37	14.24	14.23
		P2	9.79		97.91		56.76		15.12	
		P3	8.21		93.34		50.90		13.34	
1800	20	P1	9.88	9.49	95.54	95.68	55.95	54.22	14.67	14.49
		P2	9.39		97.65		56.18		15.14	
		P3	9.19		93.87		50.54		13.65

**Table 13:** Experimental data of surface roughness analysis for friction stir processed AA7075-T651/CFA/Ti-6Al-2Sn-2Zr-2Mo-2Cr-0.25 Si hybrid composites.

AA7075-T651/CFA/Ti-6Al-2Sn-2Zr-2Mo-2Cr-0.25 Si										
Rotational speed (rpm)	Processing speed (mm/min)	Point of measurement	Ra (μm)	Mean Ra (μm)	Ry (μm)	Mean Ry (μm)	Rz (μm)	Mean Rz (μm)	Rq (μm)	Mean Rq (μm)
1200	20	P1	5.62	5.37	60.38	54.40	26.33	25.29	7.56	7.62
		P2	4.98		41.90		23.46		6.74	
		P3	5.51		60.93		26.09		8.56	
1500	20	P1	5.46	5.42	59.74	55.43	24.99	24.66	7.67	7.38
		P2	4.83		45.46		23.65		6.99	
		P3	5.97		61.08		25.34		7.47	
1800	20	P1	5.12	5.15	58.36	52.85	25.41	24.71	7.15	7.37
		P2	4.52		41.04		22.85		6.08	
		P3	5.80		59.17		25.88		8.87

**Table 14:** Experimental data of surface roughness analysis for friction stir processed AA7075-T651/PKSA/Ti-6Al-2Sn-2Zr-2Mo-2Cr-0.25 Si hybrid composites.

AA7075-T651/PKSA/Ti-6Al-2Sn-2Zr-2Mo-2Cr-0.25 Si										
Rotational speed (rpm)	Processing speed (mm/min)	Point of measurement	Ra (μm)	Mean Ra (μm)	Ry (μm)	Mean Ry (μm)	Rz (μm)	Mean Rz (μm)	Rq (μm)	Mean Rq (μm)
1200	20	P1	2.38	2.50	16.84	17.52	11.95	12.09	2.87	3.17
		P2	2.92		19.79		12.57		3.99	
		P3	2.21		15.93		11.76		2.67	
1500	20	P1	2.34	2.62	16.33	17.39	11.87	12.09	2.98	3.21
		P2	2.80		19.98		12.92		3.83	
		P3	2.73		15.87		11.49		2.84	
1800	20	P1	2.26	2.27	16.56	16.97	11.14	11.56	2.82	3.00
		P2	2.48		19.24		12.12		3.57	
		P3	2.06		15.11		11.43		2.61	

## 2 Experimental design, materials and methods

The parent material used in this data collection was high strength aluminium alloy (AA7075-T651) which was received in a dimension of 500 × 600 × 6 mm^3^, this was later sectioned into required processing sizes of 300 × 125 × 6 mm^3^ using Bosch professional GDC 120 with tungsten carbide circular saw blade. The spectrometric analysis of the base metal – Aluminium Alloy 7075 – T651 is depicted in Table 1 and the various mechanical properties of AA7075 – T65, namely elongation, tensile, hardness, fatigue, modulus etc are reported in Table 2.

### 2.1 Experimental procedure and methodology for friction stir processing

**Fig. 1:**
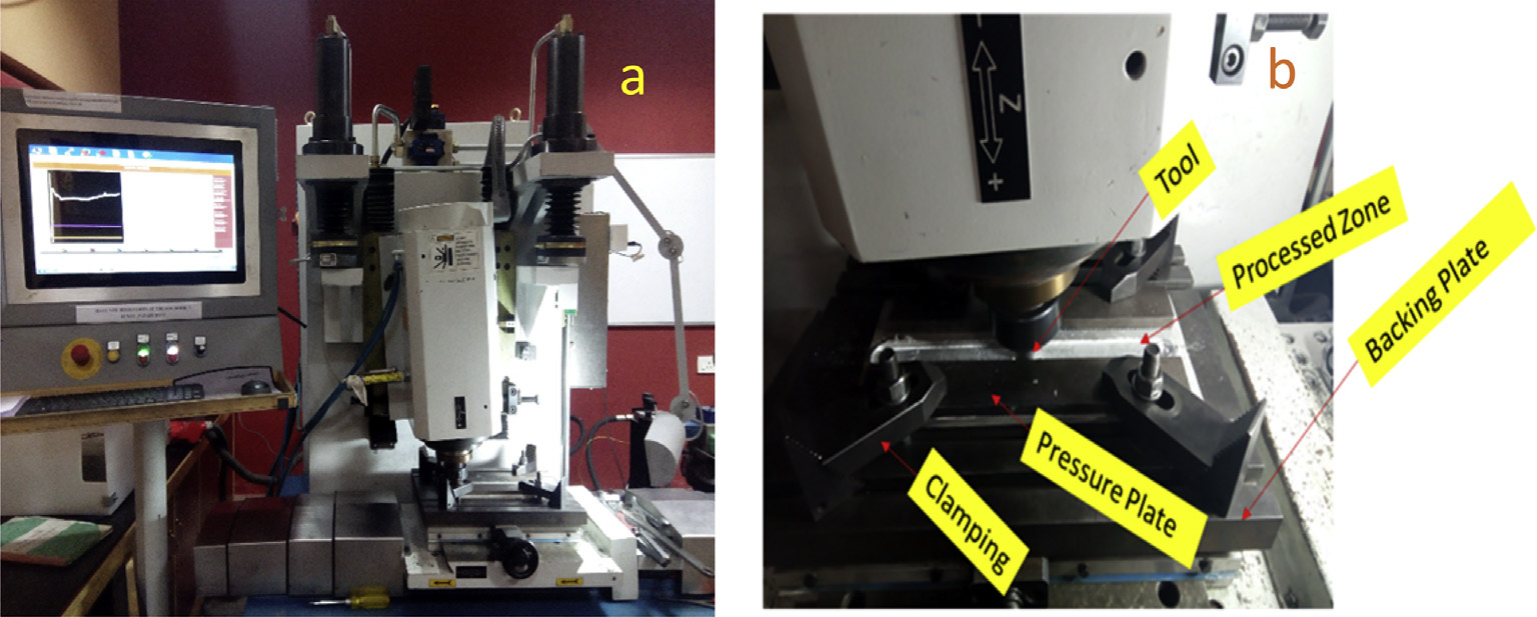
a: FSW machine showing force feedback on the Screen; b: Representation of the set –up Machine.

**Fig. 2:**
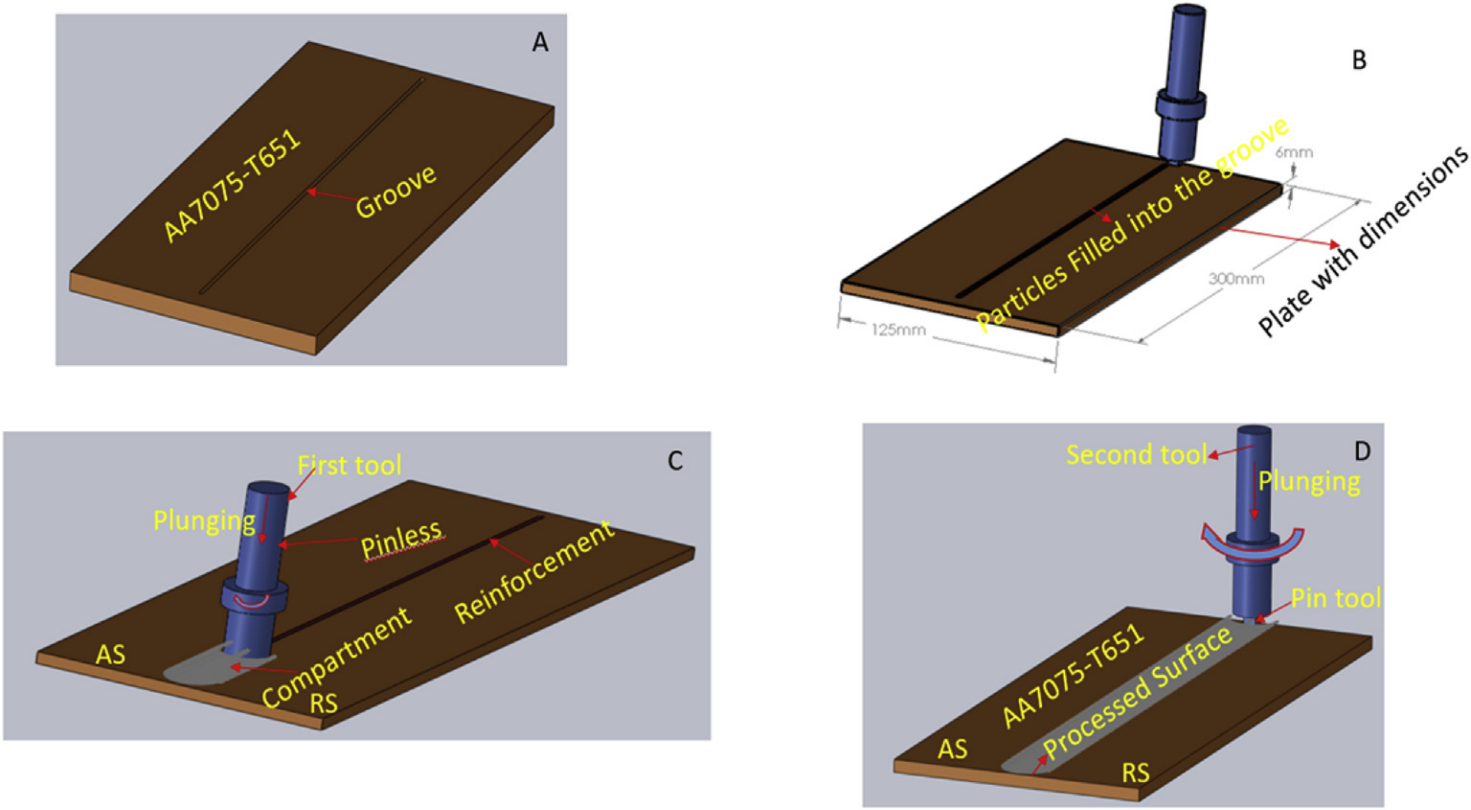
(a): Groove (b) Reinforcement (c) Compartment with pinless (d) Processed with tool with pin.

**Fig. 3:**
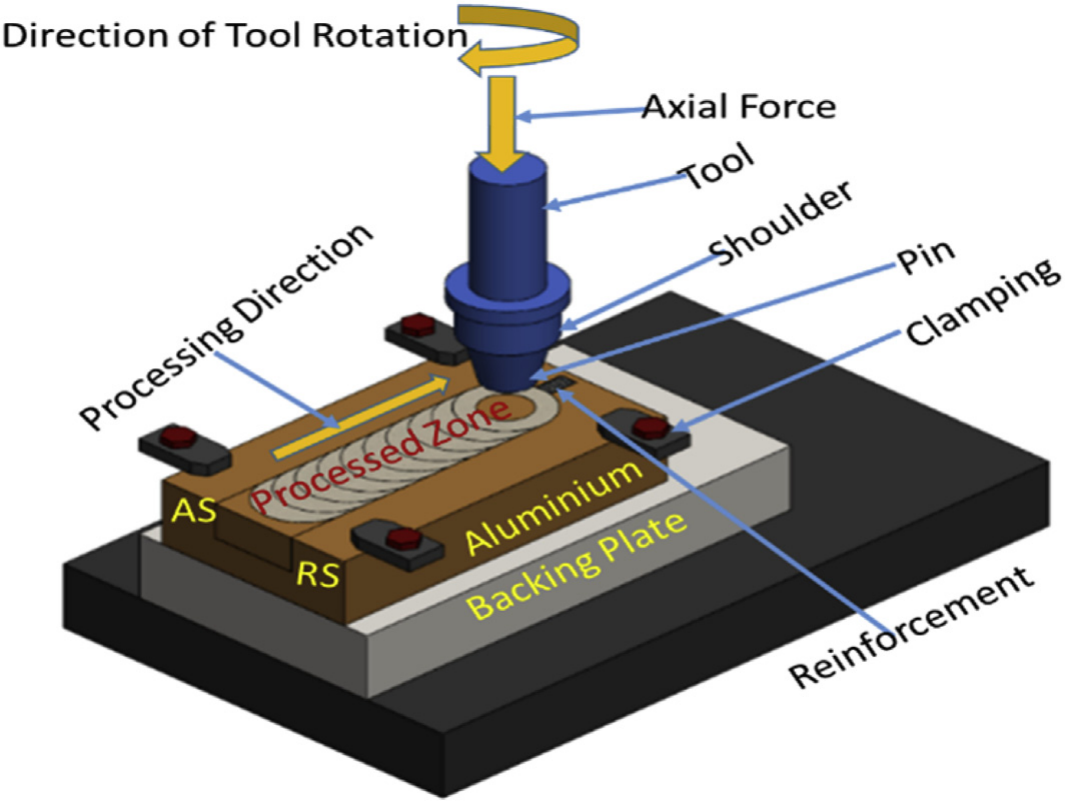
Schematic of FSP showing Advancing and Retreating Side.

Friction stir processing was conducted on a 2 Ton linear NC controlled Friction Stir Welding Machine manufactured by ETA Bangalore, India Ltd as depicted in Fig. 1a and the schematic representation of the arrangement is shown in Fig. 1b. A load cell is integrated into the machine to record forces along Z direction. Several integrated sensors also incorporated into the machine to record velocity along x –axis, x – position of the tool, z – position, x – load etc. The machine is incorporated with a LabVIEW software for acquiring data in real-time. The measurement data for the vertical axial load from the load cell was then extracted and used for the processing. Aluminium Alloy 7075-T651 of 6 mm thickness was processed with various reinforcement. Horizontal milling machine was employed to make a microchannel of 3.5 mm depth and 2.0 mm width into the plate as shown in Fig. 2a. The reinforcement particle was then packed into the groove (microchannel) as depicted in Fig. 2b. Two different tools made of AISI H13 tool steel were used in this experiment. The Pinless tool of shoulder diameter 18 mm and length 25 mm for powder compartment as depicted in Fig. 2c; and the cylindrical tapered pin tool with 18 mm shoulder diameter, 5 mm pin diameter and 5 mm pin height with 10° taper were used for stirring and processing as shown in Fig. 2d. The schematic representation of the experimental procedure is as shown in Fig. 2. Friction stir processing (FSP) is carried out on Friction stir welding (FSW) machine by positioning the aluminium plate that is being processing in a manner showing advancing side (AS) and retreating side (RS) as indicated in Fig. 3 and also placing the pressure plates (Fig. 1b) on top on Al-plate AA7075-T651 that is being worked on to enable rigid clamping and to ensure proper stirring and mixing of the materials and finishing by rotation and translation movement of the tool. The tilt of the tool towards trailing direction ensured that the shoulder of the tool held the stirred material by a cylindrical tapper pin and moved material effectively and efficiently from the front to the back of the pin. The amount of the penetration of a pin depth and tool shoulder radius contacting the workpiece is decided by the pin length. Pin profile design criteria are helpful in avoiding processes with the excessive flash, inner channel, local thinning and surface groove of the processed plates. The calculations of the proportion of the groove to the second phase materials are as shown in Eqs. (1), (2), (3) [5].

(1) $$\mathrm{Volume}\;\mathrm{of}\;\mathrm{Fraction}=\;\frac{\mathrm{Area}\;\mathrm{of}\;\mathrm{groove}}{\mathrm{Projected}\;\mathrm{Area}\;\mathrm{of}\;\mathrm{tool}\;\mathrm{pin}}\;\times \;100$$

(2) Areaofthegroove=Groovewidth×Goovedepth

(3) ProjectedAreaofthetoolpin=Pindiameter×Pinlength

### 2.2 Processing parameters

In the experiment, two different processing parameters were used. They are variable process parameters and constant process parameters. The variable process parameters used was rotational speed of 1200, 1500 and 1800 rpm with constant processing parameter of 20 mm/min processing speed, tilt angle of 3°, double passes were observed in all the processes, 0.3 mm plunge depth was achieved which was distributed thus, 0.2 mm first pass and 0.1 mm second pass. Good surface finish was achieved due to carefully selected processing parameters. The processing parameters used workpiece experimental is as showed in Table 3.

### 2.3 Surface roughness analysis

**Fig. 4:**
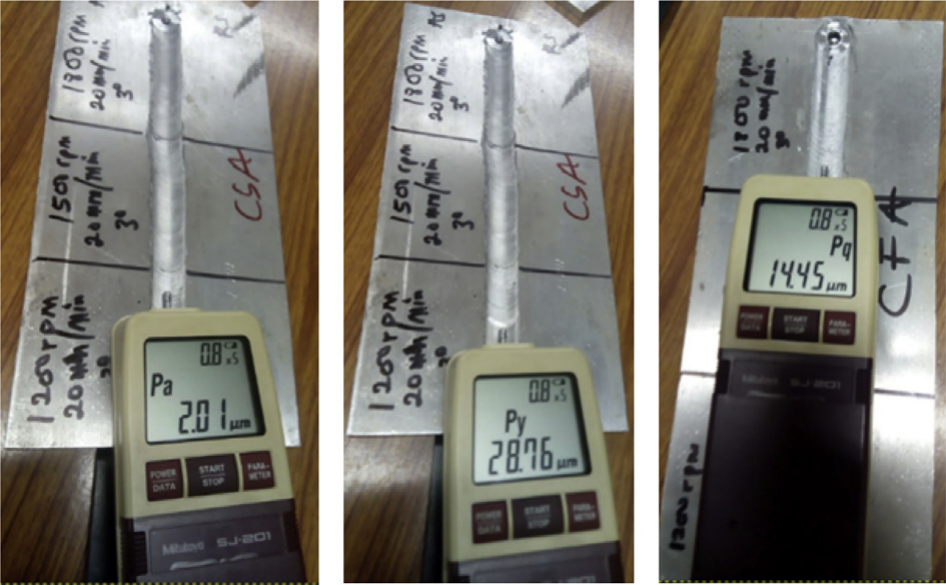
Display of surface roughness test.

**Fig. 5:**
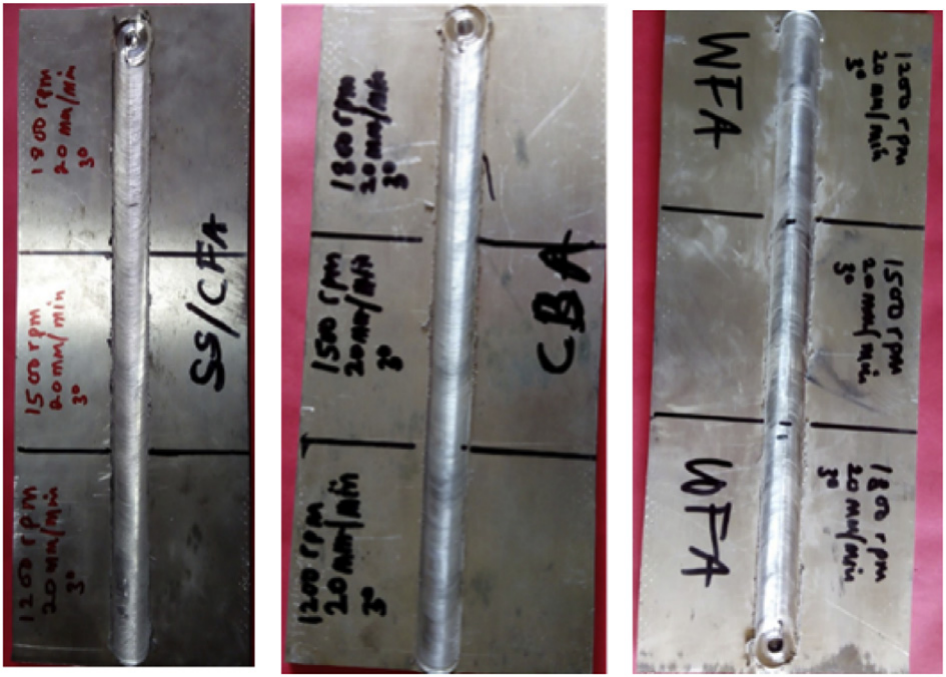
Macrographs of some processed plates.

Surface roughness analysis is a key factor to evaluate process integrity and the quality of any processed material will be determined by surface topography, hardness test [6], wear [7], microstructural analysis etc. In this present data collection, surface roughness analysis has been the key factor of the investigation. Surface integrity analysis was carried out with the aid of mitutoyo surftest SJ-210 surface roughness tester (SRT). The analysis was carried out at three different points on a parameter for a particular workpiece and the average reading for each parameter is calculated in order to ensure precision of the measurements and the coverage surface area. The following surface roughness parameters were measured and recorded; arithmetical mean roughness value (Ra), maximum height (Ry), mean roughness depth (Rz) and root mean square roughness (Rq). The experimental data recorded are presented in Table 4, Table 5, Table 6, Table 7, Table 8, Table 9, Table 10, Table 11, Table 12, Table 13, Table 14. This research therefore aimed at investigating the surface roughness obtained in friction stir processed composites and correlate it with force feed. The macrographs indicating the points of measurements on the SRT are display in Fig. 4 and samples of the measured plates are display in Fig. 5.

### 2.4 Material removal rate

The material removal rate (MRR) in friction stir processing operation is said to be the volume at which material or metal that is being worked on is removed during processing operation per unit time in mm^3^/s. This may be in form of flash or material loss in the case of reinforcements. Material is being removed or loss for each revolution of the tool. Surface roughness is determined by material removal rate. If lots of flash is observed during FSP, is then mean that the surface of the workpiece may be rough but if less or no flash is observed during processing operation, there is a tendency of having good surface finish which must be controlled by processing parameters. The material removal rate in mm^3^/s can be calculated as depicted in Eq. (4) [8]:

(4) $$MRR=\;\frac{\frac{\pi }{4}D_{0}^{2}L-\;\frac{\pi }{4}D_{i}^{2}L}{\frac{L}{FR_{s}}}$$

where, D_0_ = Initial Diameter of the workpiece (before processing) in mm, Di = Final Diameter of the workpiece (after processing) in mm, L = Length of the workpiece to be processed in mm, R_s_ = Rotational Speed in rpm and F = feed rate in mm/rev.

### 2.5 Force feedback in friction stir processing of AA7075-T651 aluminium metal composites

**Fig. 6:**
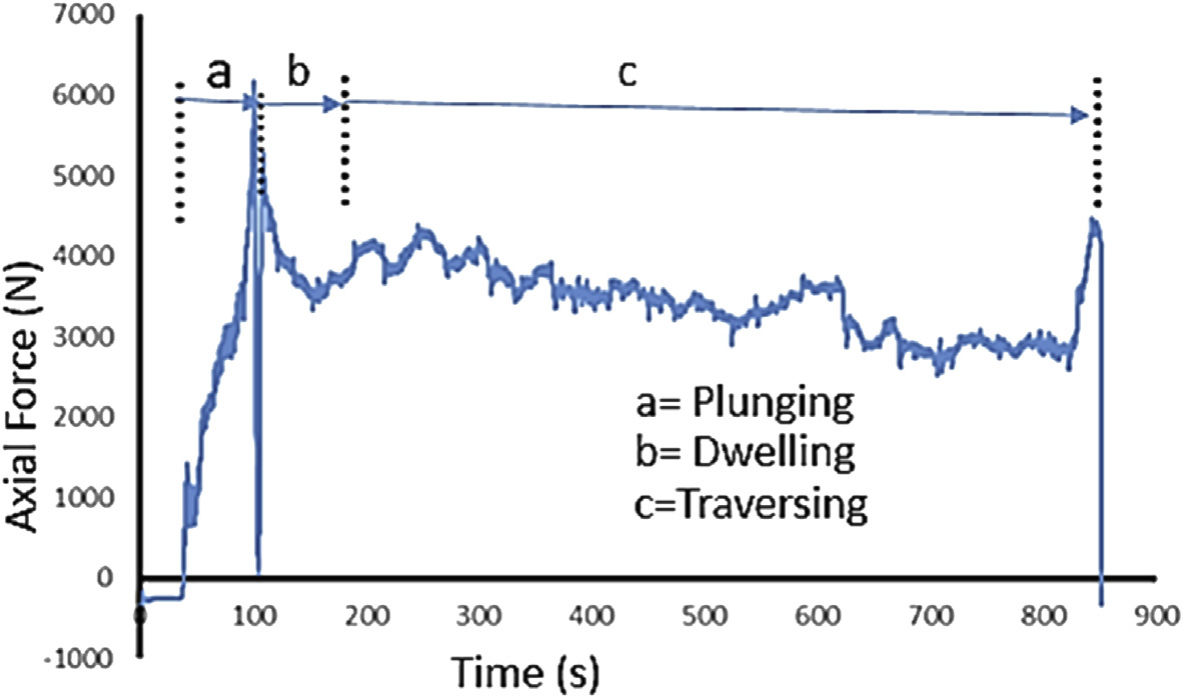
FSP feedback diagram showing (a) Plunging (b) Dwelling and (c) Traversing Stages.

**Fig. 7:**
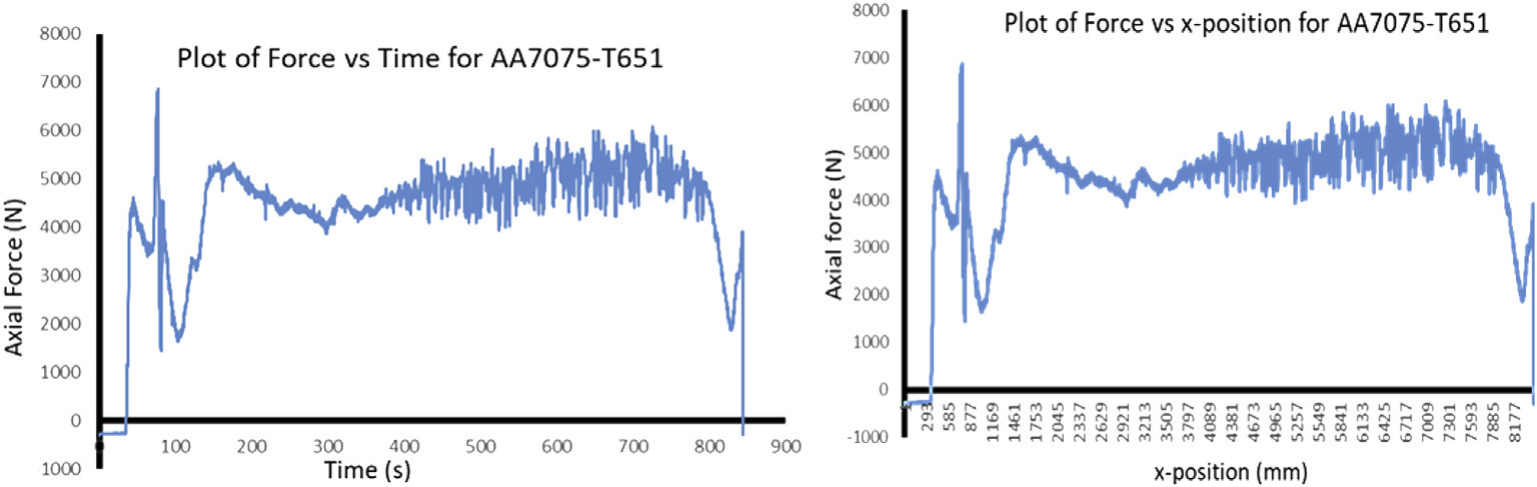
Force Feedback Variation (a) Plot of force vs time for the processed based material AA7075-T651 without reinforcement (b) Plot of force vs x-position for the processed based material AA7075-T651 without reinforcement.

**Fig. 8:**
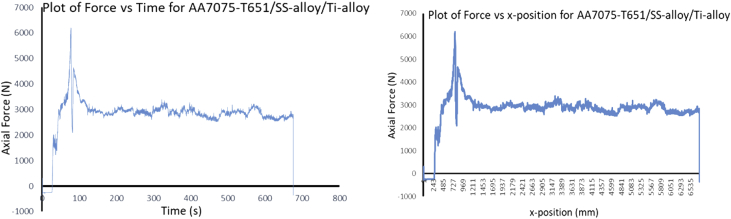
Force Feedback Variation (a) Plot of force vs time for matrix composite of stainless steel (17-4Ph)/titanium alloy (Ti-6Al-2Sn-2Zr-2Mo-2Cr-0.25 Si) reinforced processed AA7075-T651 (b) Plot of force vs x-position for matrix composite of stainless steel (17-4Ph)/titanium alloy (Ti-6Al-2Sn-2Zr-2Mo-2Cr-0.25 Si) reinforced processed AA7075-T651.

**Fig. 9:**
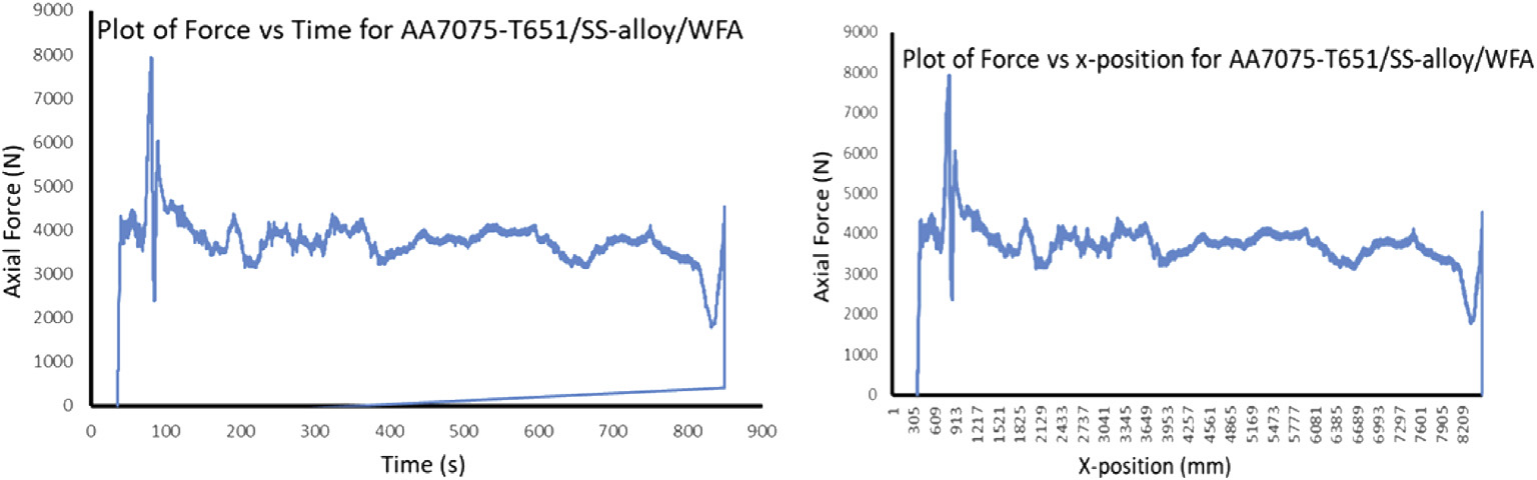
Force Feedback Variation (a) Plot of force vs time for matrix composite of stainless steel (17-4Ph)/WFA reinforced processed AA7075-T651 (b) Plot of force vs x-position for matrix composite of stainless steel (17-4Ph)/WFA reinforced processed AA7075-T651.

**Fig. 10:**
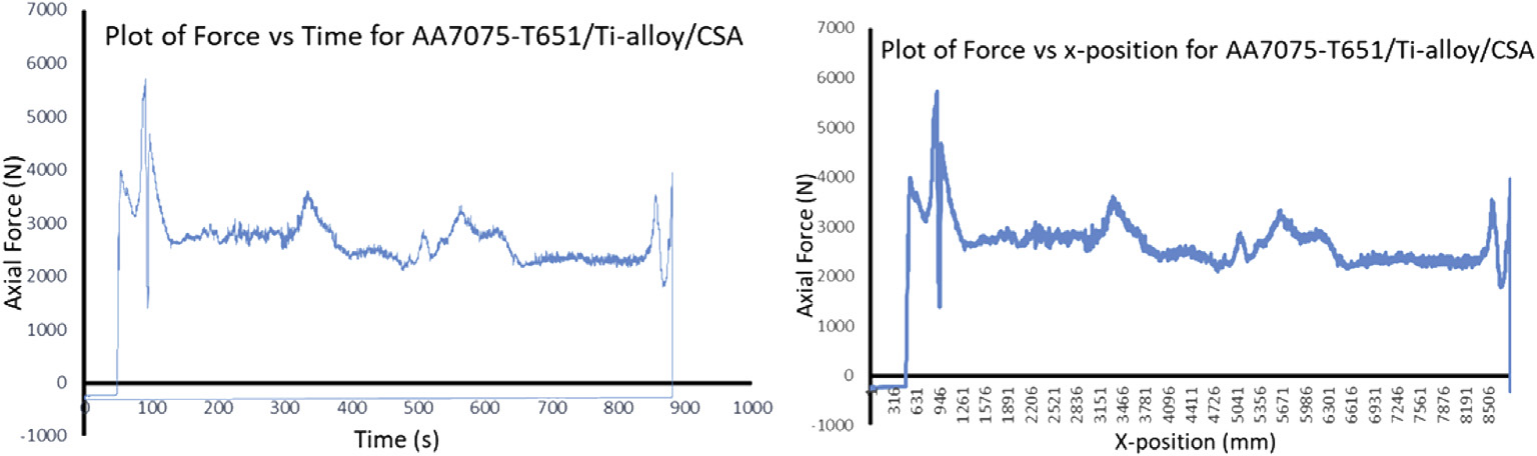
Force Feedback Variation (a) Plot of force vs time for matrix composite of titanium alloy (Ti-6Al-2Sn-2Zr-2Mo-2Cr-0.25 Si)/CSA reinforced processed AA7075-T651 (b) Plot of force vs x-position for matrix composite of titanium alloy (Ti-6Al-2Sn-2Zr-2Mo-2Cr-0.25 Si)/CSA reinforced processed AA7075-T651.

**Fig. 11:**
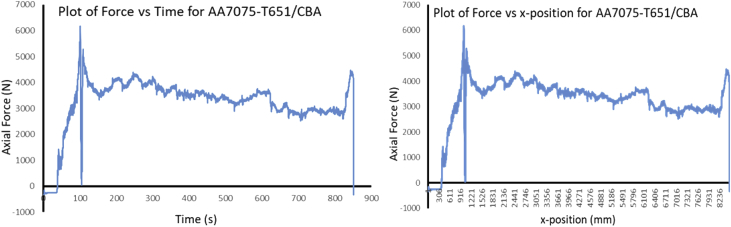
Force Feedback Variation (a) Plot of force vs time for cow bone ash (CBA) reinforced processed AA7075-T651 (b) Plot of force vs x-position for cow bone ash (CBA) reinforced processed AA7075-T651.

**Fig. 12:**
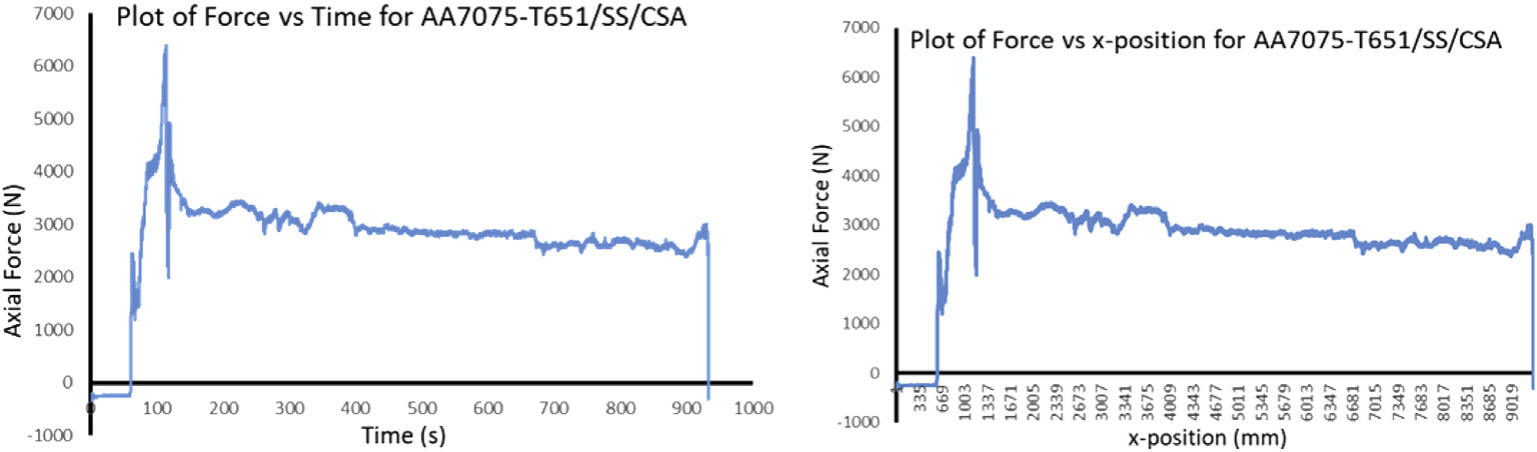
Force Feedback Variation (a) Plot of force vs time for matrix composite of stainless steel (17-4Ph)/CSA reinforced processed AA7075-T651 (b) Plot of force vs x-position for matrix composite of stainless steel (17-4Ph)/CSA reinforced processed AA7075-T651.

**Fig. 13:**
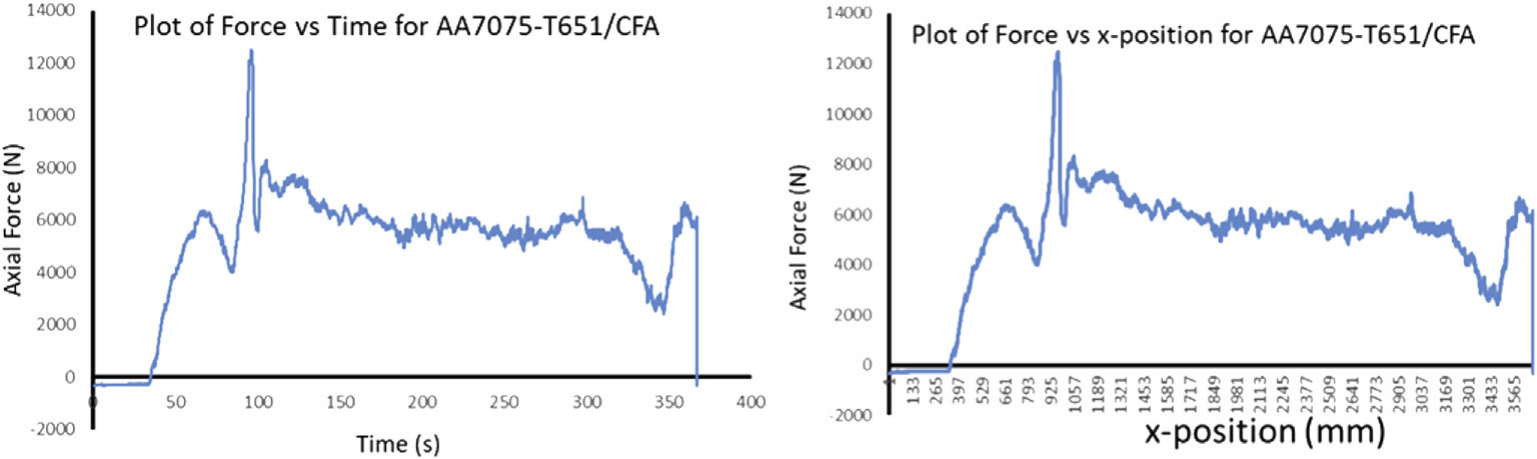
Force Feedback Variation (a) Plot of force vs time for coal fly ash (CFA) reinforced processed AA7075-T651 (b) Plot of force vs x-position for coal fly ash (CFA) reinforced processed AA7075-T651.

**Fig. 14:**
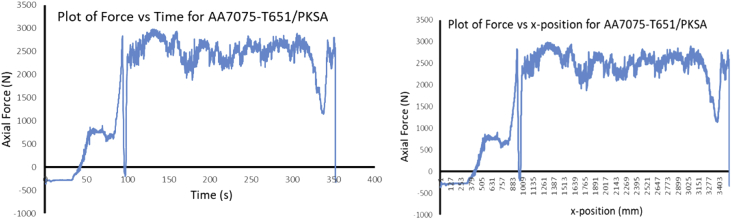
Force Feedback Variation (a) Plot of force vs time for palm kernel shell ash (PKSA) reinforced processed AA7075-T651 (b) Plot of force vs x-position for palm kernel shell ash (PKSA) reinforced processed AA7075-T651.

During FSP, the force (axial load) was measured by the FSW machine (Fig. 1a) with the aid of inbuilt piezo-electric dynamometer device. A load cell is integrated into the machine to record forces along Z direction. The feedback data from axial load (force) is very essential as this affects the machine structure and tool geometry during processing. The processing force depends on some parameters which are processing speed as well as rotational speed [9]. There are three well known operational stages during FSP, they are, plunging, dwelling and traversing as depicted in Fig. 6. During plunging, the processing tool comes in contact with the workpiece and makes a penetration into the workpiece until the shoulder of the processing tool interacts with the workpiece, a sudden jump in the axial load (force) was observed as a result of the resistance force offered by the workpiece. During the plunging of the shoulder into the workpiece, a peak load (force) is noticed and when the plunging was completed, a short dwell time (rotation of the tool at a position) was observed on the workpiece by interacting with the tool which heated up the material by friction between the shoulder of the tool and the workpiece. This heat generated led to the softening of the workpiece and the force (axial load) decreases to nearly 40% of the peak load. During traversing stage, a relative linear motion on the workpiece along the processing tool rotational motion was observed, during this traversing processing stage, a steady value of an axial load (force) was attained during further processing. Force feedback from the machine data during the friction stir processing of this work has showcased five reinforcement variations of axial forces as presented in Fig. 7, Fig. 8, Fig. 9, Fig. 10, Fig. 11, Fig. 12, Fig. 13, Fig. 14.

Surface roughness analysis is of key to manufacturing/production industries that deal with surface finish of the final product [10] especially in processing field in order to ascertain the surface integrity of the product for marketability, achieving high quality in terms of workpiece dimensional accuracy, ensuring less flash for economy of the processing in terms of cost saving, give simple mean of finisher for the product and increasing the performance of the product. The ability to control the process for better quality of the final product is of paramount importance. In FSP the surface roughness is usually depend on certain variables such the processing speed, feed rate, rotational speed, axial force feed, radial and axial depth.

It is pertinent to note, that rough surfaces usually have higher friction coefficients and wear more quickly than smooth surfaces. Fig. 7, Fig. 8, Fig. 9, Fig. 10, Fig. 11 indicate that the surface of the processed workpieces are smooth as shown on the graphs. The results from the graphs will help the users to know the behaviour of the selected aluminium alloy AA7075-T651 when interacted with different reinforcement particles as shown on the graphs.
